# Wake-Up Receiver for Underwater Acoustic Communication Using in Shallow Water

**DOI:** 10.3390/s23042088

**Published:** 2023-02-13

**Authors:** Jan H. Schmidt, Aleksander M. Schmidt

**Affiliations:** Faculty of Electronics, Telecommunications and Informatics, Gdańsk University of Technology, ul. Narutowicza 11/12, 80-233 Gdańsk, Poland

**Keywords:** underwater acoustic communication, wake-up receiver, broadband modulated signal

## Abstract

A data frame transmitted over the underwater acoustic channel usually begins with a preamble. Therefore, underwater communication systems have a dedicated receiver that constantly listens to the preamble signals. A receiver that is to work effectively in shallow waters must have solutions that effectively reduce the impact of the permanently occurring multipath propagation. The article presents a solution based on complementary broadband signals. Initial tests were carried out using the Watermark simulator to determine its reliability in such a difficult propagation environment. The results of experimental tests carried out in a model pool are also included. Details of the implementation of the wake-up receiver are presented.

## 1. Introduction

The receiver of an underwater acoustic communication system usually synchronises the transmitted data frame based on a preamble. The signals forming the preamble must effectively reduce the influence of unfavourable factors in the communication channel. In a shallow water channel, multipath propagation is an extremely important factor. It causes the receiver to receive signals from the direct path and those obtained as a result of reflections. Another important factor is the Doppler effect, which has a much greater effect in underwater acoustic systems than in terrestrial communications using electromagnetic waves. The low propagation speed of acoustic waves and the low bandwidth of transducers limit the capacity of the hydroacoustic channel [1,2]. In addition to the difficulties presented previously, it should be mentioned that some of them are characterized by seasonal variability, which significantly changes the operation of hydroacoustic systems [3,4].

Currently developed underwater acoustic communication systems are mainly based on the spread spectrum technique, the orthogonal frequency-division multiplexing (OFDM) technique, chaotic signals, as well as incoherent and coherent modulations [5,6,7,8,9,10,11]. Moreover, such systems may use a multi-antenna technique [12,13]. The most popular representatives of the spread spectrum technique are frequency-hopping spread spectrum (FHSS), direct-sequence spread spectrum (DSSS), and chirp spread spectrum (CSS), although there are also several variations [14,15,16,17,18,19,20]. Among them, there are mainly systems with solutions that ensure data transmission with the highest possible transmission rate in vertical channels and a relatively lower rate in horizontal channels. These communication systems use a data frame synchronisation system to determine the arrival of a frame signal and establish the timing of the signals in the data frame. 

In the case of point-to-point transmission, these operations are performed by a dedicated wake-up low-power receiver that continuously listens to the signals of the preamble and detects their arrival. The implementation of such a receiver typically involves the use of a processor that provides low-power modes capable of performing these operations while reducing power consumption. Since the computing power needed to receive block data is much greater, the mode is changed from low power to full active only at this point.

In turn, underwater wireless sensor networks (UWSN) contain nodes that are equipped with low-power underwater acoustic communication systems. These communication systems must ensure highly reliable data transmission to enable correct and stable operation of the entire network, including such a difficult communication channel as the shallow water channel. The nodes in a UWSN are often autonomous devices of control and measurement with limited power resources, which cannot be recharged. Therefore, the communication systems include a data frame synchronisation system, i.e., *a wake-up receiver*, that is used to wake up the underwater device and has a low-complexity structure to meet the important criterion of energy efficiency. The wake-up receiver allows the node to wait for incoming traffic in sleep mode, so it can work for a relatively long period of time.

Overall, there is strong demand from both the civil and military markets for low-power receivers for underwater acoustic communication systems with very high reliability in shallow water channels.

Many existing wake-up receiver implementations are based on solutions dedicated to radio communication, which perform this task based on the analysis of the occurrence of a signal with a specific single frequency [21,22]. However, such signals are easily distorted due to the presence of frequency-selective fading in a shallow water channel, which significantly reduces the efficiency of the frame synchronisation system and, consequently, excludes the use of such a synchronisation method due to its insufficient reliability. 

Other scientists have conducted development work using a broadband signal, or LFM, but used complex reception algorithms, which required a high-class digital signal processor with a power consumption of several hundred mW [23]. A broadband signal in the form of a pseudo-random sequence was also used in a system based on a microcontroller, but with a bit shorter duration than in the proposed solution [24].

The article presents the concept of a reliable synchronisation system consisting of several pairs of broadband signals for underwater acoustic communication operating in shallow waters. The use of frequency modulated signals is conducive to the implementation of an energy-saving system and allows operation with low signal-to-noise ratio (SNR) values. The arrangement of these signals in the preamble is intended to increase the reliability of the synchronisation system. Simulation tests in the Watermark simulator were used to test the efficiency of the synchronisation system, which uses measured channel’s impulse responses in the shallow waters. The results of experimental tests carried out in the pool are also included. This concept of the synchronisation system was developed for implementation of a communication system operating in a device with limited energy resources.

## 2. Concept of the Synchronisation System

Based on the experience gained in the implementation of various underwater communication systems, the concept of a synchronization system dedicated to shallow waters was developed, which will be used by an underwater device [21,25]. The task of this device is long-term autonomous operation as a control and measurement system. The limitation of the available power forced the choice of a technical solution based on a low-power microcontroller, which will act as a system controller and a signal-processing processor.

### 2.1. Signals and Structure of Data Frame

The presented synchronisation system is to ensure reliable communication in a shallow water channel exhibiting the multipath phenomenon, which appears constantly. The signal components reach the receiver via two or more propagation paths. They have different amplitudes and phases, which cancel out and amplify the interfering signals. The phenomenon impacts the frequency domain, where selective fading is noticed on some of the band frequencies used [26,27,28]. Thus, on a channel with selective fading, the signal phase and amplitude at the indicated frequency will be continually changing, and a synchronisation system based on the analysis of the occurrence of a signal on a single frequency is very unreliable.

In order to overcome such problems in the communication channel, a wideband hyperbolic frequency modulated (HFM) signal was used. Such signals are used in other underwater acoustic systems because they work well with low signal-to-noise ratios and are highly resistant to the Doppler effect [29,30].

In the presented synchronisation system, there is an assumption that for the HFM signal, pulses appear as two types of signals: those with an increasing frequency (HFM-UP) and those with a decreasing frequency (HFM-DOWN), which can be described by the below Equations (1) and (2).

(1) $$s_{HFM}\left(t\right)=\exp\left[-j2\pi \;\frac{\ln\left(kt+\frac{1}{f_{L}}\right)}{k}\right],$$

(2) $$\mathrm{where}\;k=\frac{f_{L}-f_{U}}{f_{L}f_{U}T_{s}}\;\;\;\mathrm{for}\;\mathrm{HFM-UP},\;\;k=\frac{f_{U}-f_{L}}{f_{L}f_{U}T_{s}}\;\;\;\mathrm{for}\;\mathrm{HFM-DOWN},\;\;$$

*f_L_*—lowest frequency, *f_U_*—highest frequency, and *T_s_*—time period of symbol signal.

**Figure 1:**
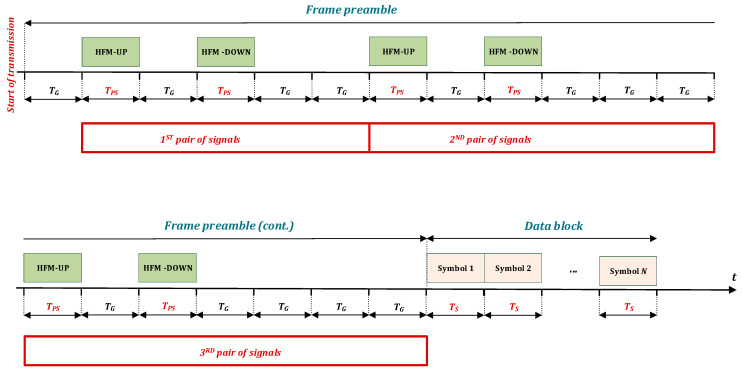
Data frame structure.

The data frame structure is composed of a frame preamble and a data block, as shown in Figure 1. The concept of the preamble structure provides for the use of several pairs of HFM-UP and HFM-DOWN signals, and in order to detect the order of a particular pair, it is assumed that different pause times are used after each pair of signals. 

These different pause times after each pair are assumed to be multiples of the *T_G_* pause time between the signals of each pair. The first pulse of the pair (HFM-UP) increases the frequency from the lowest frequency (*f_L_*) to the highest frequency (*f_U_*). The second pulse (HFM-DOWN) decreases the frequency from the highest frequency (*f_U_*) to the lowest frequency (*f_L_*). *T_PS_* is a time period of preamble HFM signals which are equal for each pair of signals. 

The purpose of using pause time *T_G_* results from the need to avoid interference between signals resulting from the reverberation phenomenon, in particular, its multipath component. The reason for the interference is the uncontrolled elongation of the pulses, and as a result, the overlapping of the preceding pulses with the next ones. The length of the pause between signals used is at least equal to the multipath delay spread *T_m_*. The condition *T_m_* ≤ *T_G_* must be met [28].

Such a preamble structure extends the preamble duration but increases the probability of correct detection of the transmitted data frame by the receiver. The proposed preamble structure enables implementation on a single-chip processor with average computational efficiency, e.g., limits the number of matched filters to be computed.

### 2.2. Detection of Synchronization Signals

**Figure 2:**
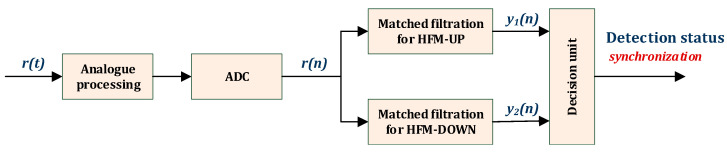
General scheme of receiving preamble signals.

Based on the preamble signals of the data frame, the real-time analysis of an incoming useful signal is carried out in the wake-up receiver. Figure 2 shows the scheme of receiving preamble signals.

During analogue filtering, the analysed signal is filtered, amplified, and shifted from the band at the centre frequency to the base band. Next, the obtained signal with a 5 kHz band is sampled according to the Nyquist criterion, i.e., with a sampling frequency of 10 kHz. Detection of the received HFM signals is performed by matched filtering. It is performed by determining a correlation function *y*(*n*) obtained by correlating a received signal *r*(*n*) and an impulse response of the matched filter *h*(*n*), that is known in the system receiver according to Equation (3).

(3) $$\;y_{1,2}\left(n\right)=\overset{M-1}{\underset{m=0}{\sum }}r\left(m\right)h\left(n-m\right)\;\;\;.\;\;$$

The interpretation of correlation functions is signal compression in the time domain. Determining a correlation function is computed for both the HFM-UP and HFM-DOWN simultaneously. 

**Figure 3:**
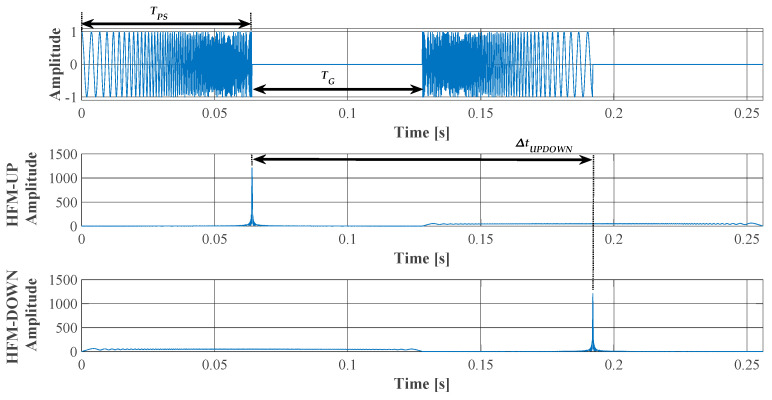
Frame preamble signals and the determined correlation functions for the received signal with the pattern of HFM-UP and HFM-DOWN signals (*T_PS_* = 64 ms, *T_G_* = 64 ms, SNR = +20 dB).

Figure 3 shows the preamble signals of the data frame *r*(*t*) based on the Equations (1) and (2) and the determined correlation functions for HFM-UP and HFM-DOWN, for *T_PS_* = 64 ms and *T_G_* = 64 ms.

The aim of using matched filtering is to enhance the receiver input signal-to-noise ratio (SNR*_r_*). The output signal-to-noise ratio of the receiver SNR*y* is determined by multiplying SNR*_r_* by product *B*·*T*, where *B* is bandwidth of the signal and *T* is an equal time period, based on Equation (4).

(4) $$\;{\mathrm{SNR}}_{y}=BT\;\cdot \;{\mathrm{SNR}}_{r}\;$$

In the receiver, the incoming signal is detected, and the receiver and transmitter synchronisation process is executed. In the data frame, a time relation is determined between the particular signals. 

**Figure 4:**
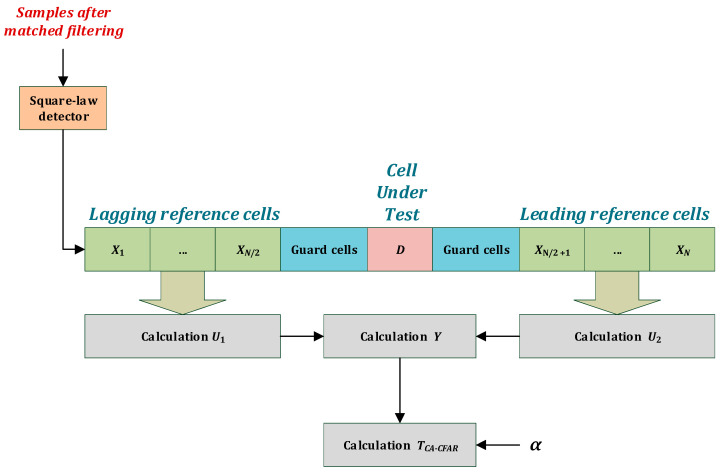
CA-CFAR block diagram.

However, the obtained correlation functions, and more precisely, the fluctuations of the correlation function maximum, i.e., the main beam, suggest the need to use an algorithm that will dynamically calculate the adaptive threshold depending on the background noise and desired false alarm rate. The Cell Averaging Constant False Alarm Rate (CA-CFAR) algorithm was chosen for this task [31]. CA-CFAR is a typical and efficient algorithm with a simple scheme. The algorithm has a relatively low computational complexity because it only determines the average level of background noise. This is desirable due to the considered hardware platform, which is a single-chip processor. The samples from the output of the square detector are fed to the input of the CFAR detector. A single threshold determination is made based on the average of reference cells’ power, surrounding the cell under test (CUT), as shown in Figure 4. There are guard cells around the CUT that are not included in the calculations. The leading *N*/2 and the lagging *N*/2 samples are the reference samples. The following sums are determined for them, according to Equation (5).

(5) $$\;U_{1}=\overset{\frac{N}{2}}{\underset{i=0}{\sum }}X_{i},\;\;U_{2}=\overset{N}{\underset{\frac{N}{2}+1}{\sum }}X_{i}\;\;\;\;.\;\;$$

Hence, the mean of *N* reference samples is calculated according to Equation (6), and is the background power level.

(6) $$\;Y=\frac{\left(U_{1}+U_{2}\right)}{2}\;\;\;.\;\;$$

The threshold can be obtained by multiplying the scale factor α with the estimated background level *Y*, as follows:

(7) $$\;T_{CA-CFAR}=\alpha \cdot Y\;\;\;.\;\;$$

The scale factor α is calculated based on the number of referenced cells (8).

(8) $$\alpha =N\;\left({P_{FA}}^{-1/N}-1\right).\;\;$$

where *N* is the number of reference cells and *P_FA_* is the probability of a false alarm. 

In the preamble of the data frame, pairs of hyperbolically modulated pulses with diverse monotonicity of the variable over time signal frequency are used. The pulses in the preamble have the same pulse duration. This configuration of a single pair of pulses ensures unequivocal estimation of the delay presumed in the transmitter between them and the Doppler shift. The fact is used that the correlation functions determined for the HFM-UP and HFM-DOWN signals are shifted in time relative to the reference function, i.e., the autocorrelation function, depending on whether the transmitter is approaching or moving away from the receiver.

Hence, the value of the Doppler shift *f_d_* can be determined from the Equation (9).

(9) $$f_{d}={\left(\frac{\Delta t_{UPDOWN}-T_{G}}{2}\right)}^{-1}\;\left[\mathrm{Hz}\right],$$

where Δ*t_UPDOWN_* denotes the time difference between the maximum of the correlation function determined for the received signal with the pattern of the transmitted HFM-UP signal, and the maximum of the correlation function for the received signal with the pattern of the HFM-DOWN signal. *T_G_* is the pause time between transmitted HFM signals in the preamble. The measurement scheme of the parameters for the calculations is presented in Figure 3. 

Such a combination of HFM-UP and HFM-DOWN signals is used to estimate the Doppler shift both in active sonars and underwater communication systems [32,33,34].

Using the correlation peaks of the frame signals obtained as a result of matched filtering and the CFAR detector, the time relationships between the peaks were determined. The method of determining pairs of HFM signals in a data frame has been written in the form of Algorithm 1.

Based on the pairs of signals used in the preamble of the data frame, and in particular the cumulative times of the gaps between them, the algorithm makes it possible to detect in the preamble the following: only the first pair of signals, only the second pair, only the third pair, the first and second pair, the second and third pair, and all signals. When parameters are determined for two or three pairs of signals in a frame, the time Δ*t_UPDOWN_* is calculated by averaging them.

The value of *λ* is a time deviation allowed in the process of determining the time relationships between correlation peaks.
**Algorithm 1.** Determination of pairs of HFM signals in a data frame*FindPair1:***if** (peak HFM-UP1)  Set *T_HFM-UP1_* = *T_0_*
 **if** (peak HFM-DOWN1 in time interval <*T_0_* + *T_G_* + *T_PS_* − λ; *T_0_* + *T_G_* + *T_PS_* + λ>)  Set *T_HFM-DOWN1_* First pair of HFM signals was detected.*FindPair2:*  **if** (peak HFM-UP2 in time interval <*T_0_* + 3*T_G_ + 2*T_PS_ − λ; *T_0_* + 3*T_G_ + 2*T_PS_ + λ>)   Set *T_HFM-UP2_*   **if** (peak HFM-DOWN2 in time interval <*T_0_* + 4*T_G_ + 3*T_PS_ − λ; *T_0_* + 4*T_G_ + 3*T_PS_ + λ>)    Set *T_HFM-DOWN2_* Two pairs of HFM signals were detected.*FindPair3:*    **if** (peak HFM-UP3 in time interval <*T_0_* + 7*T_G_ + 4*T_PS_ − λ; *T_0_* + 7*T_G_ + 4*T_PS_ + λ>)     Set *T_HFM-UP3_*     **if** (peak HFM-DOWN3 in time interval <*T_0_* + 8*T_G_ + 5*T_PS_ − λ; *T_0_* + 8*T_G_ + 5*T_PS_ + λ>)      Set *T_HFM-DOWN3_* Three pairs of HFM signals were detected (1, 2, 3).      Set the time offset to the data, *T_DATA_* = *T_HFM-DOWN3_* + 4*T_G_.
     **else**
      Two pairs of HFM signals were detected (1, 2).       Set the time offset to the data, *T_DATA_* = *T_HFM-DOWN2_* + 8*T_G_ + 2*T_PS_.
     **end**

    **else**
     Two pairs of HFM signals were detected (1, 2).      Set the time offset to the data, *T_DATA_* = *T_HFM-DOWN2_* + 8*T_G_ + 2*T_PS_.
    **end**

   **else**
    First pair of HFM signals was detected (1).     Set the time offset to the data, *T_DATA_* = *T_HFM-DOWN1_* + 11*T_G_ + 4*T_PS_.
   **end**

  **else**
    First pair of HFM signals was detected (1).     Set the time offset to the data, *T_DATA_* = *T_HFM-DOWN1_* + 11*T_G_ + 4*T_PS_.
  **end**

 **else**
  First pair of HFM signals not detected.*FindPair2 Else:*  **if** (peak HFM-UP2 in time interval <*T_0_* + 3*T_G_ + 2*T_PS_ − λ; *T_0_* + 3*T_G_ + 2*T_PS_ + λ> )   Set *T_HFM-UP2_*   **if** (peak HFM-DOWN2 in time interval <*T_0_* + 4*T_G_ + 3*T_PS_ − λ; *T_0_* + 4*T_G_ + 3*T_PS_ + λ> )    Set *T_HFM-DOWN2_* Second pair of HFM signals was detected (2).*FindPair3 Else:*    **if** (peak HFM-UP3 in time interval <*T_0_* + 7*T_G_ + 4*T_PS_ − λ; *T_0_* + 7*T_G_ + 4*T_PS_ + λ> )     Set *T_HFM-UP3_*     **if** (peak HFM-DOWN3 in time interval <*T_0_* + 8*T_G_ + 5*T_PS_ − λ; *T_0_* + 8*T_G_ + 5*T_PS_ + λ> )      Set *T_HFM-DOWN3_* Third pair of HFM signals was detected (2, 3).      Set the time offset to the data, *T_DATA_* = *T_HFM-DOWN3_* + 4*T_G_.
     **else**
      Second pair of HFM signals was detected (2).       Set the time offset to the data, *T_DATA_* = *T_HFM-DOWN2_* + 8*T_G_ + 2*T_PS_.
     **end**

    **else**
     Two pairs of HFM signals were detected (2).      Set the time offset to the data, *T_DATA_* = *T_HFM-DOWN2_* + 8*T_G_ + 2*T_PS_.
    **end**

   **else**
    First and second pair of HFM signals not detected.*FindPair3 Else2:*    **if** (peak HFM-UP3 in time interval <*T_0_* + 7*T_G_ + 4*T_PS_ − λ; *T_0_* + 7*T_G_ + 4*T_PS_ + λ> )     Set *T_HFM-UP3_*     **if** (peak HFM-DOWN3 in time interval <*T_0_* + 8*T_G_ + 5*T_PS_ − λ; *T_0_* + 8*T_G_ + 5*T_PS_ + λ> )      Set *T_HFM-DOWN3_* Third pair of HFM signals was detected (3).      Set the time offset to the data, *T_DATA_* = *T_HFM-DOWN3_* + 4*T_G_.
     **else**
      No HFM signal detected. 
     **end**

    **else**
     No HFM signal detected. 
    **end**

   **end**

  **end**

 **end**
**else** No HFM-UP signal detected. **end**

In conclusion, the detection of signals in the data frame is based on the analysis of the correlation function values obtained for the HFM-UP and HFM-DOWN patterns. The multipath phenomenon causes that the maximum values of the correlation function obtained as a result of matched filtration are burdened with fluctuations, and the form of the correlation function has a single peak or several. In fact, it is a channel impulse response (CIR), and the richness of the peaks obtained in the response depends on it.

In the next step, the CA-CFAR algorithm eliminates the influence of background noise changes on the threshold, which is calculated in an adaptive manner. The correlation functions obtained in this way from both processing paths are subject to a final algorithm that detects pairs of signals and effectively determines the reference times in the data frame.

The use of several pairs of complementary HFM signals in the preamble allows the capacity to increase the probability of their detection. In turn, the use of different time intervals between pairs of signals allows for their precise determination in the frame preamble and allows for the high precision of the reference times. In order to avoid interference between the signals, the pause of appropriate duration *T_G_* is used, the value of which should satisfy the inequality *T_G_* ≥ *T_m_*.

All of the operations used have an impact on the suppression of multipath phenomenon.

## 3. Simulation Tests

Simulation tests were carried out to determine the impact of the unfavourable factors present in the underwater channel on the reception quality of the transmitted preamble signals. First, tests were performed on a channel with the additive white Gaussian noise to determine the range of changes calculated by the correlation function, for different SNR values. Other tests were performed in an underwater channel with fading using a watermark simulator. The simulation tests were performed in MATLAB R2022 b.

### 3.1. Performance in Channel with the Additive White Gaussian Noise (AWGN)

During the tests, correlation functions were determined for HFM signals transmitted over a communication channel with additive white Gaussian noise (AWGN), in which there was no multipath propagation.

It should be noted that noise other than additive white Gaussian noise can be taken into account in the simulation testing process [35,36,37]. This applies in particular to warm shallow waters, for which snapping shrimp dominate the ambient noise spectrum above a few kHz. The results of analyses of communication systems for channels with such noise are presented in the other papers [38,39,40].

**Figure 5:**
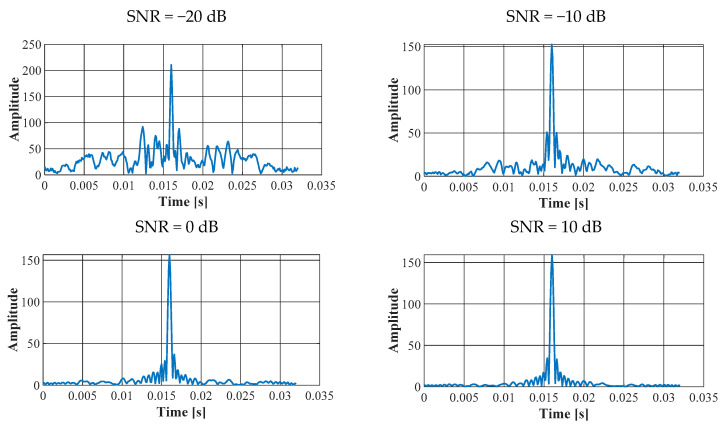
Correlation functions for the HFM-UP signal (*T_PS_* = 16 ms).

Figure 5 shows the determined correlation functions for the HFM-UP pulse and the following SNR values −10 dB, 0 dB, 10 dB and 20 dB. A single HFM-UP pulse was transmitted over the AWGN channel with a *T_PS_* duration of 16 ms, a band *B* of 5 kHz, and a Doppler shift of 0 Hz.

The correlation functions determined for various values of AWGN were consistent with the dependence (4). Hence, for a signal received in the presence of additive white Gaussian noise, the signal-to-noise ratio is proportional to the energy of the received signal and inversely proportional to the noise power spectral density. Since a broadband signal is used, the calculated main beam of the correlation function is narrow and the side lobes are low. The width of the main beam can be reduced by increasing the bandwidth *B* of the signal.

However, the maximum value of the correlation function is proportional to the duration of the signal, i.e., to the number of samples per duration. In turn, the repeatability of the calculated maximum values of the correlation function is higher when the received signal has a higher SNR. By increasing the duration of the signal or the bandwidth of the signal B, the effect of noise on the calculated value of the correlation function is reduced.

The above conclusions were derived from the simulation tests carried out, which consisted in determining the correlation functions of the received signal and the pattern of the HFM-UP signal. These tests concerned transmission over a channel with additive Gaussian noise with signal duration *T_PS_* = 1 ms, 4 ms, 8 ms, 16 ms, 32 ms and 64 ms, for several SNR values. Each calculation was repeated 10^3^ times for the selected set of parameters. The obtained ranges of maximum values of the correlation function for different *T_PS_* signal durations and SNR values are showed in Table 1.

Comparing the results of the obtained ranges for a specific signal duration *T_PS_*, for SNR = 10 dB and 20 dB, small differences between them are observed. On the other hand, for SNR = −10 dB and 0 dB, the differences between the determined range values are significant, and much larger in relation to the values obtained for SNR = 10 dB and 20 dB. These differences are also visible in the case of *T_PS_*, for which the product *B·T* takes large values, i.e., 80, 160, 320.

The obtained results show the need to use effective signal processing, allowing for operation in a wide range of SNR values.

### 3.2. Performance in Underwater Channel with Fading—Watermark Simulator

A number of simulation tests were carried out to study the influence of multipath propagation and the Doppler effect on the transmission quality of preamble signals in an underwater channel. For this purpose, the Watermark simulator was used, which is a freely available benchmark for underwater acoustic communications [41]. The benchmark is a shell around the validated channel simulator Mime that uses impulse responses measured in the sea [42,43]. The simulator convolves user signals with the measured channel’s impulse response. This operation is expressed as follows:

(10) $$y\left(t\right)=\int_{-\infty }^{\infty }\hat{h}\left(t,\tau \right)x\left(t-\tau \right)d\tau +n\left(t\right),$$

where *x*(*t*) is the input user signal, $\hat{h}\left(t,\tau \right)$ time-varying impulse response, *n*(*t*) noise, and *y*(*t*) output signal. The single-input single-output (SISO) configuration was used during the tests.

Two communication channels that were included in the simulator and made available as impulse responses measured in Norway-Oslofjord (NOF1) and Norway-Continental Shelf (NCS1) were used. These impulse responses were recorded between a stationary projector and a stationary receiving hydrophone placed on the bottom. The used parameters of the Watermark channels and the signal are presented in Table 2.

The NOF1 channel is a shallow area of the Oslofjorden inlet, and it is characterised by relatively stable arrivals. In turn, the NCS1 channel has no stable subsequent arrivals. The channel multipath parameter in the form of the multipath delay spread *T_m_* for both channels is similar and amounts to approximately 12 ms [28].

The generated preamble signals for different durations *T_PS_* (4 ms, 8 ms, 16 ms, 32 ms and 64 ms) were sent by the NOF1 and NCS1 channels in the presence of additive Gaussian noise at different SNR values (−10 dB, −3 dB, 0 dB, 10 dB). 

**Figure 6:**
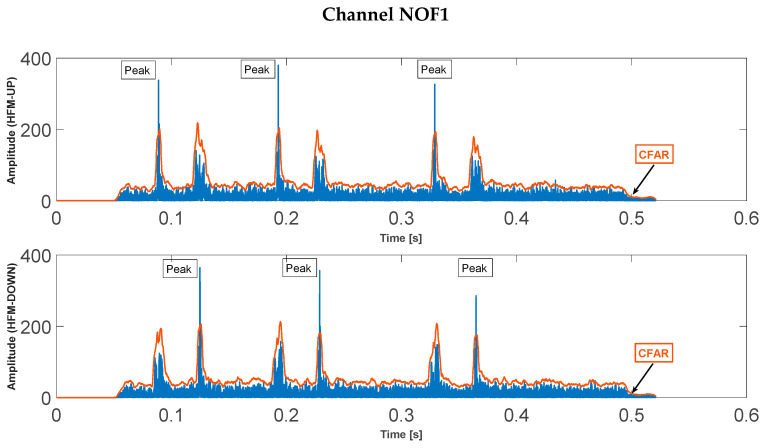
Correlation functions for the received signal on channel NOF1 (SNR *=* −3 dB, *T_PS_* = 4 ms).

**Figure 7:**
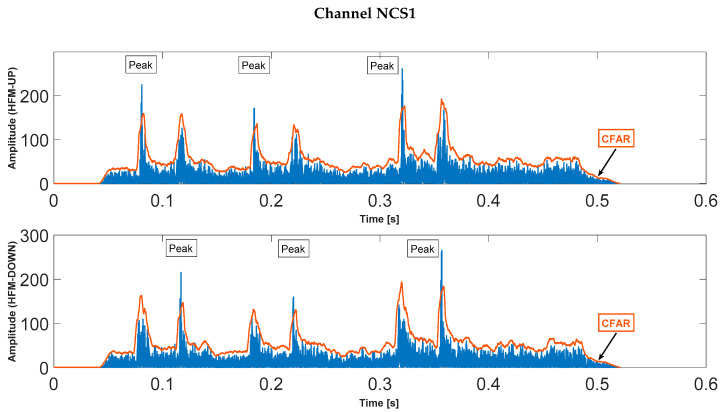
Correlation functions for the received signal on channel NCS1 (SNR *=* −3 dB, *T_PS_* = 4 ms).

Selected results of the calculations of the correlation function and the result of the CFAR detector, for both channels are shown in Figure 6 and Figure 7.

For the calculated correlation function, a CFAR detector and an algorithm for determining the time relationships between the signal pairs in the preamble were applied, and then the Preamble Error Rate (PER) was determined.

PER represents the ratio of the number of preamble frames detected in error to the number of all preamble frames sent. For PER1, only the first pair of preamble signals were considered for detection. Two pairs of preamble signals are included for PER2 and three pairs of preamble signals for PER3.

The results of the determined Preamble Error Rate (PER) values for different values of *T_PS_* and SNR are shown in Table 3 for channel NOF1 and Table 4 for channel NCS1.

The obtained results indicate that the reception of the preamble signals is error free for the three pairs of signals, and both the NOF1 and NCS1 channels, regardless of the SNR.

In the NOF1 channel, the considered synchronisation system with a single and a double pair of HFM signals confirmed its high reliability for all *T_PS_* and SNR values of 0 dB and 10 dB. There were no preamble reception errors for *T_PS_* of 32 and 64.

In the non-stationary channel, which is the NCS1 channel, the operation of the synchronisation system with a large *BT* product enables error-free transmission, i.e., for one pair of HFM signals and *T_PS_* = 64 ms, and for a double pair of HFM signals and *T_PS_* = 32 ms, 64 ms. The use of three pairs also enabled error-free reception for all *T_PS_*.

In order to compare the PER values obtained for the considered method, the PER values were determined for the case when the threshold is set to a constant value of 50% of the maximum value of the correlation function for the selected *T_PS_* time. Results are shown in Table 5 for channel NOF1 and Table 6 for channel NCS1.

The presented tables indicate that the use of the determination of pairs of HFM signals in a data frame algorithm with the CFAR algorithm allowed the obtaining of better results.

The obtained results confirm the fact that the NCS1 channel is more demanding than NOF1 [44]. For NCS1, there were large fluctuations of the determined value of the correlation function.

## 4. Experimental Tests

**Figure 8:**
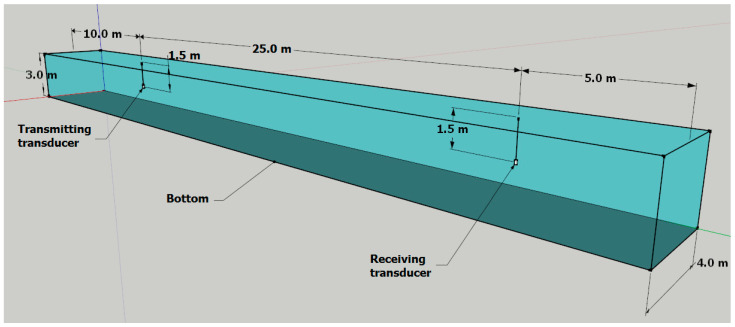
Dimensions of the model pool and the location of transmitting and receiving transducers.

Experimental tests were carried out in a model pool of the Gdańsk University of Technology, Poland, with the following dimensions: 40 m long, 4 m wide, and 3 m deep. The bottom and walls of the pool are made of concrete, without any diffusing or absorbing material. The arrangement of the transmitting and receiving transducers is shown in Figure 8. The multipath delay spread *T_m_* for this channel amounts to approximately 35 ms. Such a large value of Tm is caused by multiple reflections on the model pool boundaries.

The same measuring apparatus was used to carry out both tests, both for the transmitting and the receiving parts. Computers running the MATLAB environment were used to generate the transmitted signals and analyse the received signals. 

In turn, the signals were subjected to both analogue-to-digital and digital-to-analogue sampling using an NI-USB6363 Multifunction I/O Device from National Instruments. Underwater transmission was provided by HTL-10 underwater telephones by Sonel with ultrasonic transducers, which are currently used by the Polish Navy on Mi-14 helicopters [21]. In transmitting mode, the underwater telephone receives an analogue signal, which is then amplified and sent to the transmitting transducer. In receiving mode, the HTL-10 receives the signal from the receiving transducer, which is filtered and then sampled by the NI-USB6363 to obtain samples. Both the transducers are omnidirectional with a resonant frequency of 34 kHz.

**Figure 9:**
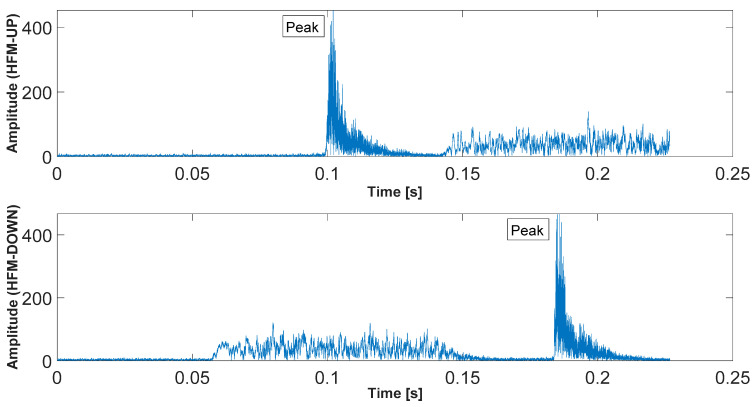
Correlation functions for the signal pair HFM-UP and HFM-DOWN (*T_PS_* = 64 ms).

The generated preamble signals for different *T_PS_* durations (4 ms, 8 ms, 16 ms, 32 ms, and 64 ms) were sent between the transducers. The selected results of calculations of the correlation function for the received signal pair HFM-UP and HFM-DOWN are shown in Figure 9. The duration of the *T_G_* pause was set at 32 ms.

The figures reveal that, as in the stimulation tests, there is not a single peak of the correlation function here, but rather a long split. This reduces the maximum value of the correlation function, which is then subjected to the next steps of reception.

The determined values of the Preamble Error Rate (PER) for different values of *T_PS_* are presented in Table 7.

As in Section 3, in order to compare the PER value for the considered method, the rightmost column contains the values obtained for the threshold set to a fixed value of 50% of the maximum value of the correlation function for the selected *T_PS_* time.

The results indicate that in such a communication channel for signals with a long *T_PS_* duration, i.e., 64 ms, and thus a high *BT* product, error-free reception was obtained regardless of the number of signal pairs used. For signals with a shorter duration (lower *BT* product value), the splitting of the main-beam calculated correlation function reduces the possibility of detecting the signal. For proper operation in a difficult communication channel, it is required to use signal durations, i.e., 16–64 ms.

In summary, the PER results prove the high reliability of the synchronisation system with an increase in the used pairs of broadband HFM signals.

## 5. Implementation of Wake-Up Receiver

Existing wake-up receivers working on a radio channel usually constantly listen for a narrowband pilot signal, and after detecting such a signal, feed it to the system block responsible for receiving the signal containing data [45]. 

For underwater channels, communication systems have been developed in which wake-up receivers are patterned on solutions from radio communication, including for radio-frequency identification RFID [45]. Wake-up receivers are implemented on the basis of discrete analogue circuits [21] or integrated circuits, such as the popular ultra-low-power AS3933 integrated circuit [46]. This device allows for the detection of a specific frequency or the correlation of received data with a pre-set pattern. The data are extracted via an On-Off-Keying (OOK) modulated carrier envelope. The advantage of this system is its very low power consumption in analysis mode, amounting to a dozen μW. In turn, the bandwidth of signals received by the system is 15–150 kHz. The lower frequency range for some applications is too high and insufficient to be used. The first reason for using lower frequencies is to provide a long-range communication system. The second concerns underwater devices placed on the bottom, which can be buried in the bottom sediments or intentionally placed in them. They usually require the use of an acoustic wave with a frequency lower than 15 kHz [47]. 

However, the use of a narrowband signal as a pilot signal in an underwater communication system designed for operation in shallow waters with severe fading is an ineffective and unreliable solution. 

In order to increase the operational reliability of the underwater acoustic communication system, the use of a broadband HFM signal was assumed, and a wake-up receiver is the key element of the system, which must ensure its reliable detection on such a difficult data transmission channel. This requires the development of a receiver using a microcontroller or processor that will perform the necessary calculations in real-time with relatively low power consumption. The interpretation of the obtained correlation functions makes it possible to determine the start of the data frame preamble and the subsequent data in the frame. 

The receiver to detect the preamble signals of the data frame determines the incoming signal cross-correlation functions separately for the HFM-UP and HFM-DOWN signal. The correlation functions are determined based on the obtained digital signals that have previously undergone analogue processing and analogue-to-digital conversion. Analog processing consists of band filtering the received signal, amplifying it, and converting it from a band around the centre frequency to a base-band. As a result of these analogue processing operations, a 5 kHz base-band signal is obtained, which is then sampled according to the Nyquist criterion, i.e., at a sampling rate of 10 kHz, and the real samples are obtained. Therefore, for a pulse with duration *T_s_* = 8 ms, there are 80 samples, and for 16 ms it is 160 samples.

At the beginning of the selection of the platform for the implementation of digital signal processing algorithms, the use of field-programmable gate arrays and signal processors was rejected, considering that they do not meet the requirements of low power consumption.

Therefore, the MSP430 FR5994 microcontrollers (MCUs) from Texas Instruments were selected. They are low-power microcontrollers that provide fast performance for digital signal processing with a low-energy accelerator (LEA). The LEA module is a 32-bit hardware engine capable of performing digital signal processing operations without the participation of a processor, and after their completion, it generates an interruption. It can perform both 16-bit and 32-bit fixed-point operations in both real and complex domains.

The available *Digital Signal Processing (DSP) library for MSP microcontrollers* (MSP-DSPLIB) provides easy-to-use APIs that allow convenient use of the LEA module by programming in a high-level environment. This library is available free of charge and contains many useful functions needed to perform fixed-point signal processing operations. The APIs manage the LEA module and apply optimal configurations to the LEA registers [48].

The use of the LEA module is possible only in active or low-power LPM0 mode, in which the microcontroller CPU is turned off and the FRAM memory is in standby mode. Both modes use the main MCLK timing signal that is required for the LEA module [49].

The MSP430 FR5994 MCU has 8 kB of SRAM, half of which is shared with the LEA for input, output, data parameters, and the main application. The processor architecture used allows for independent operation of both the CPU and the LEA module.

In the presented underwater communication system, the receiver performs calculations on real samples, which significantly reduces the amount of computational power that would be required in the case of complex numbers.

The cross-correlation of two real continuous functions, *y_xh_* is defined by Equation (11):

(11) $$\;y_{xh}\left(t\right)=\int_{-\infty }^{+\infty }x\left(\tau -t\right)h\left(\tau \right)d\tau \;\;\;,\;\;$$

while the convolution can be written as:

(12) $$\;x\left(t\right)*h\left(t\right)=\int_{-\infty }^{+\infty }x\left(t-\tau \right)h\left(t\right)d\tau \;\;\;.\;\;$$

The only difference between them is that in the case of cross-correlation, one of the two functions is not reversed. Thus,

(13) $$\;y_{xh}\left(t\right)=x\left(-t\right)*h\left(t\right)\;\;\;.\;\;$$

The MSP-DSPLIB library includes an optimised FIR filter function but not the correlation function; therefore, the above feature was used to compute the cross-correlation function of the received signal with the HFM-UP and HFM-DOWN signal patterns. Its use only requires reversing of the signal pattern data.

The implementation of the FIR filter uses a discrete-time convolution, and the computational operations are performed on fixed-point numbers in the Q15 format. 

The available function allows the user to configure different sizes of the input data vector and the size of the coefficient vector. The vector size limitation is the available SRAM-shared memory size.

The considered underwater communication system requires the implementation of the necessary tasks in real-time. The analysis cycle was set to 20 ms and at a sampling rate of *f_s_* = 10 kHz, the input data vector is constant and contains *N_x_* = 200 samples in the Q15 format, and the size of the coefficient vector *N_h_* depending on the signal duration *T_s_* is shown in Table 8. The vector of coefficients was generated using the MATLAB computing environment.

The following main tasks are performed during a single analysis cycle:

- Signal sampling using the on-chip ADC12 module (200 obtained samples are transferred to shared memory using DMA);
- Determination of the cross-correlation function for the HFM-UP and HFM-DOWN patterns;
- Algorithm detection of the preamble signals.

To ensure continuity between successive cycles of analysis, the last input samples from the ADC should be retained after completing the calculations in the cycle for use in a new cycle by copying them to the beginning of the input data vector.

**Figure 10:**
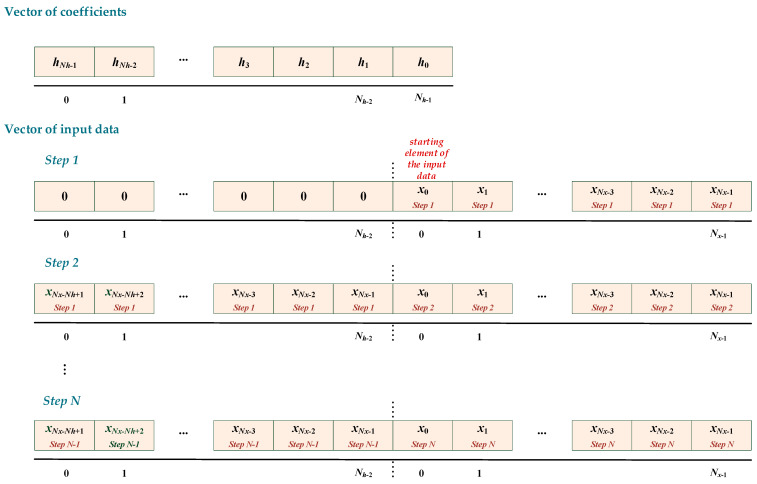
The scheme of operations on the input data subject to calculations.

The number of data stored must be equal to *N_h_* − 1. Therefore, the total size of the input buffer that is used in the calculation by the FIR function is *N_x_* + *N_h_* − 1. A general diagram explaining these operations is presented in Figure 10.

Table 9 shows the averaged measurement results of the calculation time *T_cp_* in the analysis cycle equal to 20 ms, depending on the signal duration *T_s_*. The times were obtained using an MCLK clock signal of 16 MHz. The use of MCLK = 8 MHz causes a twofold extension of the calculation time *T_cp_*. The SRAM memory is available in this model of the MSP430 FR family processor, which is shared with the LEA for input data, and was sufficient to perform calculations for the maximum required values of the vectors *N_h_* = 320 and *N_x_* = 200.

The microcontroller‘s power consumption was determined using the EnergyTrace power analyser tool, available in the Code Composer Studio development environment, dedicated to the development of Texas Instruments microcontrollers and processors. This value depends on the used clock signal MCLK, and so for MCLK = 8 MHz it was about 15 mW and for MCLK = 16 MHz about 20 mW.

Comparing the obtained values of power consumption, it should be mentioned here that the low-power signal processors of the TI C5000 series, which would be able to perform these operations, have a typical power consumption of the hundreds of mW. Another feature that adversely affects the use of the signal processor is the complex system environment around it, e.g., external FLASH memory is required. 

## 6. Conclusions

The article presents a concept of a reliable synchronisation system consisting of several pairs of broadband signals for underwater acoustic communication operating in shallow waters. The use of several pairs of these signals in the preamble was intended to increase the reliability of the synchronisation system. 

Simulation tests with the Watermark simulator and experimental tests in a model pool were performed to check the performance of the synchronisation system. The tests confirmed its reliability in such a difficult propagation environment.

The detection algorithms are implemented using a single-chip processor and provide real-time operation with low power consumption.

## Figures and Tables

**Table 1:** Ranges of maximum values of the correlation function for different values of *T_PS_* and SNR.

*T_s_*	SNR			
	−10 dB	0 dB	10 dB	20 dB
1 ms (BT = 5)	4.5–27.9	7.0–15.9	9.8–12.7	10.6–11.5
4 ms (BT = 20)	20.8–59.7	35.0–47.4	38.5–42.4	39.9–41.3
8 ms (BT = 40)	58.3–102.6	71.8–86.8	77.2–82.2	79.0–80.6
16 ms (BT = 80)	126.3–187.0	149.1–168.5	156.0–162.7	158.0–160.1
32 ms (BT = 160)	274.9–361.7	301.9–330.9	312.8–320.9	315.8–318.4
64 ms (BT = 320)	566.8–701.1	610.3–649.3	626.0–638.5	629.9–633.8

**Table 2:** Parameters of the Watermark channels and the signal [44].

	NOF1	NCS1
Environment	Fjord	Shelf
Range	750 m	540 m
Water depth	10 m	80 m
Centre frequency	14 kHz	14 kHz
Bandwidth	8 kHz	8 kHz
Doppler coverage	7.8 Hz	31.4 Hz
Type	SISO	SISO

**Table 3:** Preamble Error Rate (PER) values for different values of *T_PS_* and SNR—channel NOF1.

	*T_PS_* [ms]	SNR			
		−10 dB	−3 dB	0 dB	10 dB
PER1	4	0.116	0.052	<10^−6^	<10^−6^
	8	0.033	0.021	<10^−6^	<10^−6^
	16	0.010	<10^−6^	<10^−6^	<10^−6^
	32	<10^−6^	<10^−6^	<10^−6^	<10^−6^
	64	<10^−6^	<10^−6^	<10^−6^	<10^−6^
PER2	4	0.041	0.16	<10^−6^	<10^−6^
	8	0.017	<10^−6^	<10^−6^	<10^−6^
	16, 32, 64	<10^−6^	<10^−6^	<10^−6^	<10^−6^
PER3	4, 8, 16, 32, 64	<10^−6^	<10^−6^	<10^−6^	<10^−6^

**Table 4:** Preamble Error Rate (PER) values for different values of *T_PS_* and SNR—channel NCS1.

	*T_PS_* [ms]	SNR			
		−10 dB	−3 dB	0 dB	10 dB
PER1	4	0.317	0.172	0.065	0.023
	8	0.078	0.044	0.021	<10^−6^
	16	0.043	0.013	<10^−6^	<10^−6^
	32	0.023	0.009	<10^−6^	<10^−6^
	64	<10^−6^	<10^−6^	<10^−6^	<10^−6^
PER2	4	0.093	0.057	0.020	0.007
	8	0.031	0.022	0.003	<10^−6^
	16	0.012	<10^−6^	<10^−6^	<10^−6^
	32, 64	<10^−6^	<10^−6^	<10^−6^	<10^−6^
PER3	4, 8, 16, 32, 64	<10^−6^	<10^−6^	<10^−6^	<10^−6^

**Table 5:** Preamble Error Rate (PER) values for different values of *T_PS_* and SNR—channel NOF1 (Threshold 50%).

	*T_PS_* [ms]	SNR			
		−10 dB	−3 dB	0 dB	10 dB
PER1	4	0.227	0.135	0.047	0.014
	8	0.155	0.088	0.031	0.003
	16	0.072	0.054	0.011	<10^−6^
	32	0.029	0.013	<10^−6^	<10^−6^
	64	<10^−6^	<10^−6^	<10^−6^	<10^−6^
PER2	4	0.106	0.056	0.027	0.015
	8	0.060	0.031	0.013	0.003
	16	0.021	0.011	0.002	<10^−6^
	32	0.008	<10^−6^	<10^−6^	<10^−6^
	64	<10^−6^	<10^−6^	<10^−6^	<10^−6^
PER3	4	0.016	0.003	<10^−6^	<10^−6^
	8, 16, 32, 64	<10^−6^	<10^−6^	<10^−6^	<10^−6^

**Table 6:** Preamble Error Rate (PER) values for different values of *T_PS_* and SNR—channel NCS1 (Threshold 50%).

	*T_PS_* [ms]	SNR			
		−10 dB	−3 dB	0 dB	10 dB
PER1	4	0.408	0.211	0.137	0.067
	8	0.180	0.113	0.085	0.031
	16	0.080	0.073	0.052	0.015
	32	0.037	0.028	0.025	<10^−6^
	64	0.016	0.009	0.008	<10^−6^
PER2	4	0.143	0.101	0.081	0.056
	8	0.091	0.072	0.051	0.015
	16	0.053	0.040	0.032	0.008
	32	0.024	0.022	0.010	<10^−6^
	64	0.004	0.003	0.001	<10^−6^
PER3	4	0.041	0.021	<10^−6^	<10^−6^
	8	0.005	0.002	<10^−6^	<10^−6^
	16, 32, 64	<10^−6^	<10^−6^	<10^−6^	<10^−6^

**Table 7:** Preamble Error Rate (PER) values for different values of *T_PS_*.

	*T_PS_* [ms]	PER	PER
			Threshold 50%
PER1	4	0.831	0.861
	8	0.533	0.627
	16	0.093	0.581
	32	0.031	0.423
	64	<10^−6^	0.151
PER2	4	0.795	0.882
	8	0.442	0.652
	16	0.023	0.323
	32	<10^−6^	0.137
	64	<10^−6^	0.074
PER3	4	0.771	0.876
	8	0.477	0.541
	16	<10^−6^	0.231
	32	<10^−6^	0.057
	64	<10^−6^	0.004

**Table 8:** Size of the coefficients vector depending on the signal duration.

*T_s_* [ms]	*N_h_*
4	40
8	80
16	160
32	320

**Table 9:** Averaged calculation times *T_cp_* versus signal duration *T_s_*, obtained using the clock signal MCLK = 16 MHz.

*T_s_* [ms]	*T_cp_* [ms]
4	4.5
8	6.4
16	8.2
32	14.2

## Data Availability

Not applicable.

## References

[1] Lurton X. (2010). An Introduction to Underwater Acoustics: Principles and Applications.

[2] Etter P.C. (2018). Underwater Acoustic Modeling and Simulation.

[3] Katsnelson B., Petnikov V., Lynch J. (2012). Fundamentals of Shallow Water Acoustics.

[4] Klusek Z., Lisimenka A. (2016). Seasonal and diel variability of the underwater noise in the Baltic Sea. J. Acoust. Soc. Am..

[5] Dol H.S., Casari P., van der Zwan T., Otnes R. (2017). Software-Defined Underwater Acoustic Modems: Historical Review and the NILUS Approach. IEEE J. Ocean. Eng..

[6] Xu T., Xu L. (2016). Digital Underwater Acoustic Communications.

[7] Zhou S., Wang Z. (2014). OFDM for Underwater Acoustic Communications.

[8] Kochanska I., Schmidt J.H., Marszal J. (2020). Shallow water experiment of OFDM underwater acoustic communications. Arch. Acoust..

[9] Bai C., Ren H.-P., Grebogi C., Baptista M.S. (2018). Chaos-Based Underwater Communication With Arbitrary Transducers and Bandwidth. Appl. Sci..

[10] Bai C., Ren H.P., Baptista M.S., Grebogi C. (2019). Digital underwater communication with chaos. Commun. Nonlinear Sci. Numer. Simul..

[11] Karimov T., Rybin V., Kolev G., Rodionova E., Butusov D. (2021). Chaotic Communication System with Symmetry-Based Modulation. Appl. Sci..

[12] Schmidt J.H., Schmidt A.M., Kochanska I. Multiple-Input Multiple-Output Technique for Underwater Acoustic Communication System. Proceedings of the 2018 Joint Conference Acoustics.

[13] Kim S., Yoo Y. (2017). MIMO-HFM: A MIMO System with Hyperbolic Frequency Modulation for Underwater Acoustic Communication. Wirel. Pers. Commun..

[14] Schmidt J.H. (2020). Using Fast Frequency Hopping Technique to Improve Reliability of Underwater Communication System. Appl. Sci..

[15] Kochanska I., Salamon R., Schmidt J.H., Schmidt A.M. (2021). Study of the Performance of DSSS UAC System Depending on the System Bandwidth and the Spreading Sequence. Sensors.

[16] Kaminsky E. (2006). Chirp signaling offers modulation scheme for underwater communications. SPIE Newsroom.

[17] Su Y., Liu X., Jin Z., Fu X. Fast Estimation of Underwater Acoustic Multipath Channel Based on LFM Signal. Proceedings of the Global Oceans 2020: Singapore—U.S. Gulf Coast.

[18] Pecci A.C., Laot C., Bourre A. Quadratic chirp modulation for underwater acoustic digital communications. Proceedings of the OCEANS 2015.

[19] Lee J., An J., Ra H.-I., Kim K. (2020). Long-Range Acoustic Communication Using Differential Chirp Spread Spectrum. Appl. Sci..

[20] Ren H.-P., Bai C., Kong Q., Baptista M.S., Grebogi C. (2017). A chaotic spread spectrum system for underwater acoustic communication. Phys. A Stat. Mech. Appl..

[21] Schmidt J., Zachariasz K., Salamon R. (2005). Underwater communication system for shallow water using modified MFSK modulation. Hydroacoustics.

[22] Sánchez A., Blanc S., Yuste P., Perles A., Serrano J.J. (2012). An Ultra-Low Power and Flexible Acoustic Modem Design to Develop Energy-Efficient Underwater Sensor Networks. Sensors.

[23] Wang D., Li H., Xie Y., Hu X., Fu L. (2019). Channel-Adaptive Location-Assisted Wake-up Signal Detection Approach Based on LFM Over Underwater Acoustic Channels. IEEE Access.

[24] Yue M., Zheng Y.R., Chen Z., Han Y. Microcontroller implementation of low-complexity wake-up receiver for wireless sensor nodes in severe multipath fading channels. Proceedings of the 2016 IEEE/OES China Ocean Acoustics (COA).

[25] Schmidt J.H. (2016). The development of an underwater telephone for digital communication purposes. Hydroacoustics.

[26] Proakis J.G. (2000). Digital Communication.

[27] Simon M.K., Alouini M.-S. (2005). Digital Communication over Fading Channels.

[28] Molisch A.F. (2010). Wireless Communications.

[29] Marszal J. (2015). Experimental Study of Silent Sonar. Arch. Acoust..

[30] Yang J., Sarkar T.K. (2006). Doppler-invariant property of hyperbolic frequency modulated waveform. Microw. Opt. Technol. Lett..

[31] Richards M.A. (2022). Fundamentals of Radar Signal Processing.

[32] Wang F., Du S., Sun W., Huang Q., Su J. (2017). A method of velocity estimation using composite hyperbolic frequency-modulated signals in active sonar. J. Acoust. Soc. Am..

[33] Wei R., Ma X., Zhao S., Yan S. (2020). Doppler Estimation Based on Dual-HFM Signal and Speed Spectrum Scanning. IEEE Signal Process. Lett..

[34] Ling Z., Xie L., Chen H. Joint Doppler Scale Estimation and Timing Synchronization in Underwater Acoustic Communications. Proceedings of the IEEE International Conference on Signal Processing 2019, Communications and Computing (ICSPCC).

[35] Zhang X., Ying W., Yang P., Sun M. (2020). Parameter estimation of underwater impulsive noise with the Class B model. IET Radar Sonar Navig..

[36] Mahmood A., Chitre M. Modeling colored impulsive noise by Markov chains and alpha-stable processes. Proceedings of the OCEANS 2015.

[37] He W., Yu R., Zheng Y., Jiang T. Image Denoising Using Asymmetric Gaussian Mixture Models. Proceedings of the 2018 International Symposium in Sensing and Instrumentation in IoT Era (ISSI).

[38] Chitre M., Potter J., Heng O.S. Underwater acoustic channel characterisation for medium-range shallow water communications. Proceedings of the Oceans ’04 MTS/IEEE Techno-Ocean ’04 (IEEE Cat. No.04CH37600).

[39] Chitre M., Ong S.H., Potter J. Performance of coded OFDM in very shallow water channels and snapping shrimp noise. Proceedings of the OCEANS 2005 MTS/IEEE.

[40] Ahn J., Hojun Lee H., Kim Y., Chung J. (2020). Snapping shrimp noise detection and mitigation for underwater acoustic orthogonal frequency division multiple communication using multilayer frequency. Int. J. Naval Archit. Ocean Eng..

[41] van Walree P., Socheleau F.X., Otnes R., Jenserud T. (2017). The Watermark Benchmark for Underwater Acoustic Modulation Schemes. IEEE J. Ocean. Eng..

[42] van Walree P.A., Jenserud T., Smedsrud M. (2008). A discrete-time channel simulator driven by measured scattering functions. IEEE J. Sel. Areas Commun..

[43] Otnes R., van Walree P.A., Jenserud T. (2013). Validation of replay-based underwater acoustic communication channel simulation. IEEE J. Ocean. Eng..

[44] van Walree P., Otnes R., Jenserud T. Watermark: A realistic benchmark for underwater acoustic modems. Proceedings of the IEEE 3rd Underwater Communications and Networking Conference (UComms).

[45] Piyare R., Murphy A.L., Kiraly C., Tosato P., Brunelli D. (2017). Ultra Low Power Wake-Up Radios: A Hardware and Networking Survey. IEEE Commun. Surv. Tutor..

[46] (2015). 3D Low Frequency Wake-Up Receiver.

[47] Tesei A., Fawcett J.A., Lim R. (2008). Physics-based detection of man-made elastic objects buried in high-density-clutter areas of saturated sediments. Appl. Acoust..

[48] (2020). MSP430FR58xx, MSP430FR59xx, and MSP430FR6xx Family..

[49] (2021). MSP430FR599x, MSP430FR596x Mixed-Signal Microcontrollers.

